# Enhancement of the Mechanical Properties in Al–Si–Cu–Fe–Mg Alloys with Various Processing Parameters

**DOI:** 10.3390/ma11112150

**Published:** 2018-11-01

**Authors:** Su-Seong Ahn, Sharief Pathan, Jar-Myung Koo, Chang-Hyun Baeg, Chan-Uk Jeong, Hyoen-Taek Son, Yong-Ho Kim, Kap-Ho Lee, Soon-Jik Hong

**Affiliations:** 1 Division of Advanced Materials Engineering, Kongju National University, Cheonan-si 331-717, Korea; ssahn710@naver.com (S.-S.A.); shareefpathan2012@gmail.com (S.P.); koo5257@kongju.ac.kr (J.-M.K.); 2 Dongyang A.K Korea Co. Ltd., 3rd plant/R&D center 70, Wonhapgang 1-gil, Yeondong-myeon, Sejong 30067, Korea; rndchb@akglobal.net (C.-H.B.); rndcuj@akglobal.net (C.-U.J.); 3 Korea Institute of Industrial Technology, Gwangju, 61011, Korea; sht50@kitech.re.kr (H.-T.S.); woinm@kirech.re.kr (Y.-H.K.); 4 Department of Materials Science & Engineering, Chungnam National University, Daejeon 34134, Korea; kapho@cnu.ac.kr

**Keywords:** Al-Si alloy, extrusion, microstructure, T6 heat treatment, silicon content

## Abstract

In this research, various processing conditions were implemented to enhance the mechanical properties of Al-Si alloys. The silicon content was varied from hypoeutectic (Si-10 wt.%) to eutectic (Si-12.6 wt.%) and hypereutectic (Si-14 wt.%) for the preparation of Al-XSi-3Cu-0.5Fe-0.6 Mg (X = 10–14%) alloys using die casting. Subsequently, these alloys were hot-extruded with an optimum extrusion ratio (17:1) at 400 °C to match the output extruded bar to the compressor size. An analysis of the microstructural features along with a chemical compositional analysis were carried out using scanning electron microscope along with energy dispersive X-ray spectroscopy and transmission electron microscope. The SEM micrographs of the extruded samples displayed cracks in primary Si, and the intermetallic (β-Al_5_FeSi) phase was fragmented accordingly. In addition, the silicon phase was homogenously distributed, and the size remained constant. The mechanical properties of the extruded samples were enhanced by the increase of silicon content, and consequently the ductility decreased. By implementing proper T6 heat treatment parameters, coherent Al_2_Cu phases were formed in the Al matrix, and the Si phase was gradually increased along with the silicon content. Therefore, high tensile strength was achieved, reaching values for the Al-XSi-3Cu-0.5Fe-0.6Mg (X = 10–14%) alloys of 366 MPa, 388 MPa, and 420 MPa, respectively.

## 1. Introduction

Nowadays, the automobile and aerospace industries are fascinated by lightweight materials that possess superior mechanical properties and consume less fuel thus possibly mitigating the pollution crisis. To achieve these requirements, aluminium (Al) alloys are in a primary position, having additional properties such as high specific strength, good corrosion resistance, low coefficient of thermal expansion, and high strength-to-weight ratio [1,2,3,4,5]. Aluminium alloys with a high weight percentage of silicon (Si) are widely used in the automotive industry, and Si is the alloying element that sustains the aluminium casting industry. The high volumes of silicon in Al-Si alloys can consistently enhance the alloys’ mechanical properties and fluidity, reducing cracking and improving feeding to minimize shrinkage porosity. Moreover, Al-Si alloys are ubiquitous, inexpensive, and lightweight because of the high quantity of silicon [6,7,8,9,10]. The mechanical properties of Al-Si alloys re dependent on the size, shape, and distribution of eutectic and primary silicon particles [4,6,11]. To achieve these required silicon characteristics and to refine the Al-Si matrix, hot extrusion is an effective plastic deformation technique that makes the size of silicon particles smaller and can improve their mechanical strength [11,12,13,14,15]. Srivastava et al. [14] reported that the silicon and intermetallic phases were effectively fragmented to the nano-scale, resulting in enhanced mechanical properties by the hot extrusion process. Especially, the hot extrusion process was used for the production of aluminium alloys to reduce the porosity of the alloys and produce a severe shear stress, that are beneficial for plastic deformation [11,16]. It also considers other features, like temperature, die size, and hot-extrusion ratio, which help modify the resulting microstructure to improve the mechanical properties. In most cases, to achieve better mechanical properties, a high extrusion ratio that exerts high strain and pressure was used to effectively refine the final microstructure [8,13,15,16,17,18,19].

The effective strain equation (ε) is related to the extrusion ratio as follows [16]:

(1) ε=2 lnER; ER = AcAE

where *ER*, *A_C_*, and *A_E_* are extrusion ratio, area of container, and area of extruded bar, respectively.

However, the output material from the extruded machine has to maintain the required application area to produce an effective design and to reduce scraps. Hence, the extrusion ratio is fundamental when producing automobile parts, especially compressor parts. Therefore, a combination of optimum extrusion ratio and higher mechanical properties has to be preserved to obtain high-strength compressor parts in industrial applications.

Moreover, microstructure modifications and high strength in Al-Si alloys can be achieved by adding elements (Zn, Mg, Cu, Mn, Fe, Ni, Ti) and by heat treatment [1,2,3,4]. Each doping element has their own unique properties, and heat treatment may improve the doping elements’ properties. For example, magnesium makes an alloy strong, hard, and responsive to heat treatment. Copper increases the room and high-temperature properties of an alloy by forming Al_2_Cu intermetallic phases with heat treatment. Iron provides strength, improves wear resistance and thermal stability by precipitating the intermetallic compound Al_5_FeSi, and, at low concentrations, acts as a microstructure refiner [3,4,12,20,21]. To enhance the mechanical properties, a precise heating parameter, has to be selected. T6 heat treatment was carried out by three methods: solution heat treatment, quenching, and age hardening [2,15,22]. A Cu- and Mg-containing Al-Si alloy should be maintained at precise heat-treatment conditions for the appropriate length of time for the dissolution of Cu and Mg to realize the full aging potential and to obtain a homogenous supersaturated solution. Depending upon the quenching rate, the amount of solute in the supersaturated solid solution can be decided. With a high cooling rate, higher concentrations of solute are retained in solution for better mechanical properties. By choosing precisely the aging temperature and time, it is also possible to strengthen an Al-Si alloy through the coherent precipitates [7,22].

Therefore, in this research, our aim was to produce high-strength Al-XSi-3Cu-0.5Fe-0.6Mg (X = 10–14%) alloys for compressor part production. To achieve this requisite, we changed the silicon volume percentage aiming at high strength and chose a 17:1 extrusion ratio that reduced wastage. Each Al-Si billet was plastically deformed, and T6 heat treatment was also carried out to enhance the mechanical properties. Precise T6 heat treatments were performed considering a range of aging times to improve the mechanical properties. With precise T6 heat treatment conditions and an optimum extrusion ratio, we achieved the highest tensile strength of 420 MPa for a 14 wt.% Si sample as a result of the formation of coherent Al_2_Cu phases in the Al-matrix.

## 2. Experimental Procedure

In this research, the Al-XSi-3Cu-0.5Fe-0.6Mg (X = 10, 12.6, 14 wt.%) alloys were fabricated. The parent alloys with a high purity of 99.99% were purchased from R&D Korea Pvt LTD. (Ansan, Korea) and weighed according to the stoichiometric ratio of 2 kg, with various silicon contents (10, 12.6, 14 wt.%). Initially, these alloys were placed in a graphite crucible which was kept inside a high-frequency induction furnace, and the temperature was maintained at 750 °C to obtain the molten alloy. The molten alloy was transformed into stainless steel mold and rested in air. As-cast cylindrical billets of Al-XSi-3Cu-0.5Fe-0.6Mg (X = 10, 12.6, 14 wt.%) alloys were used for further experiments. According to the Si wt.%, the alloys were differentiated as alloy A (Si-10 wt.%), alloy B (Si-12.6 wt.%), and alloy C (Si-14 wt.%), respectively. The resultant cast billets were preheated for 1 h at 400 °C, and subsequently hot extrusion was performed at 400 °C with a 500-ton extruder. The obtained extruded Al-Si bars were about 1000 mm long and 16 mm in diameter. The extrusion was carried out under the conditions of an extrusion ratio of 17:1, a ramp speed of 4.5 mm/min, and an output temperature of 410 °C. The extruded samples were subjected to T6 aging treatment with solution heat treatment at 500 °C for 5 h, quenched with hot water at 60 °C, and then artificially aged at 200 °C for different times (1, 2, 3, and 4 h). The heat treatment was carried out using an electric vacuum furnace, and the oxidation was disabled by circulating Ar gas. The characterization was carried out along parallel to the extrusion direction. The phase of the T6 heat-treated extruded specimens was characterized by using an X-ray diffractometer (XRD) with high energy monochromatic Cu–Kα radiation in the 2*θ* range from 20 to 80°, with a scan speed of 2°/min (Rigaku, MiniFlex-600, Tokyo Japan). The microstructure of the extruded samples was examined using scanning electron microscopy (SEM- MIRA-LMH II (TESKAN, Brno-Kohoutovice, Czech Republic),), and the T6 annealed specimens were probed using transmission electron microscopy (TEM, Tecnai G2 F30 S-Twin/FEI company, Hillsboro, OR, USA) operated at 200 kV. The samples for the TEM images were prepared by using a focused ion beam (Helios Nanolab 450 F1/FEI company, Hillsboro, OR, USA) equipment. EDS was carried out to examine the chemical composition of the extruded samples. Vickers hardness of the extruded samples was measured at different locations using a Vickers hardness tester with a denting load of 1 kgf for 10 s, and the values were averaged. The tensile strength of the extruded and T6 specimens were meticulously investigated for two times to obtain significant and reliable values, using ASTM A370 small-size samples (D: 4 mm, L: 20 mm) with a load cell of 7.5 KN and a crosshead speed of 2.0 mm/min. The tensile strength was measured at room temperature using a tensile testing machine (UTM-T, R&B, Daejeon, Korea). The density of the casted alloys was measured 15 times using the Archimedes principle and then averaged for better accuracy.

## 3. Results and Discussion

### 3.1. Microstructure Investigation of the Extruded Samples

**Figure 1:**
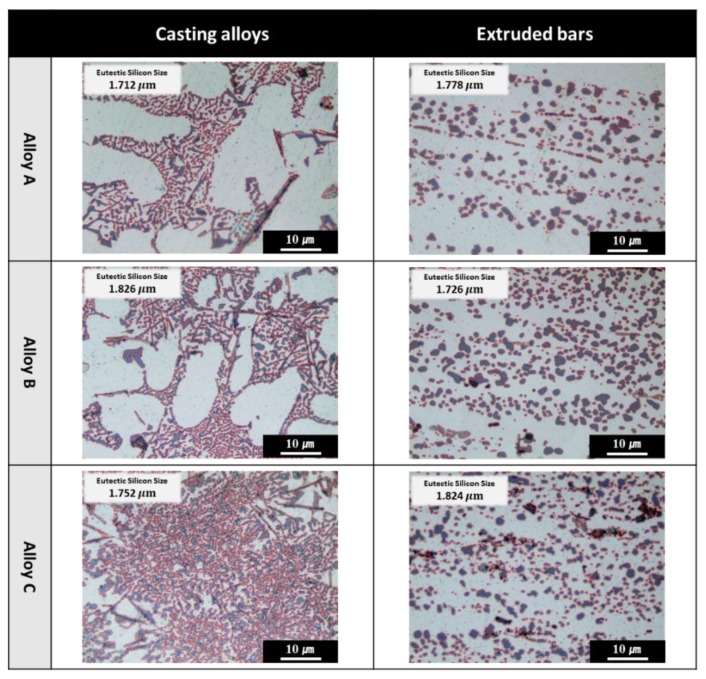
Optical micrographs and particle size analysis of silicon in both casting and extruded Al-XSi-3Cu-0.6Mg-0.5Fe [X = (alloy A): 10; (alloy B): 12.6; (alloy C): 14] alloys.

In this research, fine extruded bars without cracks were successfully fabricated. Hence, it appears that the employed extrusion parameters were able to prevent the breakage of the extruded bars. Figure 1 shows the optical micrograph images of as-cast and extruded alloys. It is clearly seen from Figure 1 that the silicon distribution in cast A, B, and C alloys was irregular, and, after extrusion, the silicon phase was well modified and evenly distributed. From the particle analysis, it was evident that the average eutectic silicon size remained almost constant in both cast and extrusion materials for alloy A, alloy B, and alloy C, which was due to the low extrusion ratio.

**Figure 2:**
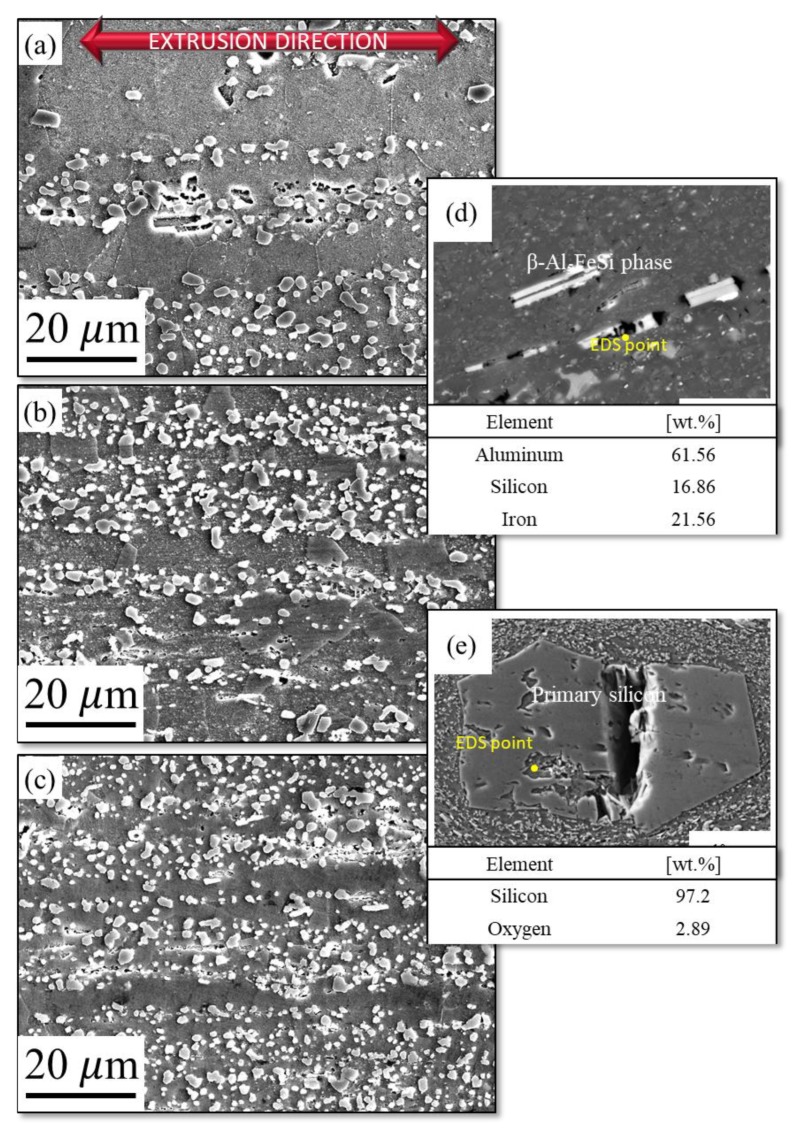
SEM Micrographs of Al-XSi-3Cu-0.6Mg-0.5Fe [X = (**a**): 10; (**b**): 12.6; (**c**): 14] extruded alloys; (**d**) β-Al_5_FeSi intermetallic compound phase and (**e**) primary silicon in Al-Si extruded bars.

Figure 2 shows the SEM micrographs for the extruded bars. From Figure 2a–c, it is apparent that, as the silicon content in the Al-Si-Cu-Fe-Mg compound increased from 10 to 14 wt.%, the Al-Si eutectic and Si phase gradually increased. It is also evident that eutectic silicon phase was homogeneously distributed in the Al matrix along the extrusion direction. This was due to the fact that the Al-Si alloy comprised soft Al and hard Si, and, when the hot-extrusion process was initiated, aluminium became softer, and the resulting deformation became uniform [6]. A similar behavior was observed by Wang et al. [13], who reported that, after hot extrusion, the silicon particles progressively matured, and their density increased. From our results, we concluded that the silicon particles were evenly distributed with the optimum extrusion ratio, and hot extrusion appeared as an optimal methodology to refine and distribute the silicon phase.

As seen in Figure 2d,e, the extruded samples contain primary Si-phase and needle shaped intermetallic compound (β-Al_5_FeSi) which was formed during the solidification process [10,21]. The compositional analysis was performed by EDS, which was shown in the inset Figure 2d,e. From EDS, it was assured that the β-phase and primary silicon were evolved in the matrix. In addition, in the inset picture of Figure 2d,e, the intermetallic (β-Al_5_FeSi) phase was fragmented consistently and a crack was appeared in the primary-Si along the extrusion direction which shows brittle fracture behavior during hot extrusion. The observed crack in the primary-Si was may be due to the extruding force and shearing stress. However, Si was not fractured well due to low extrusion ratio and this type of behavior was also reported by Zuo et al. [1].

The density value of Al-Si cast alloys is shown in Table 1. The density values slightly decreased when increasing the silicon content. Since silicon has a low density (~2.3 g/cm^3^) compared to aluminium (~2.7 g/cm^3^), the alloy’s density decreased with silicon addition.

### 3.2. Mechanical Properties of the Extruded Alloys Without T6 Heat Treatment

**Figure 3:**
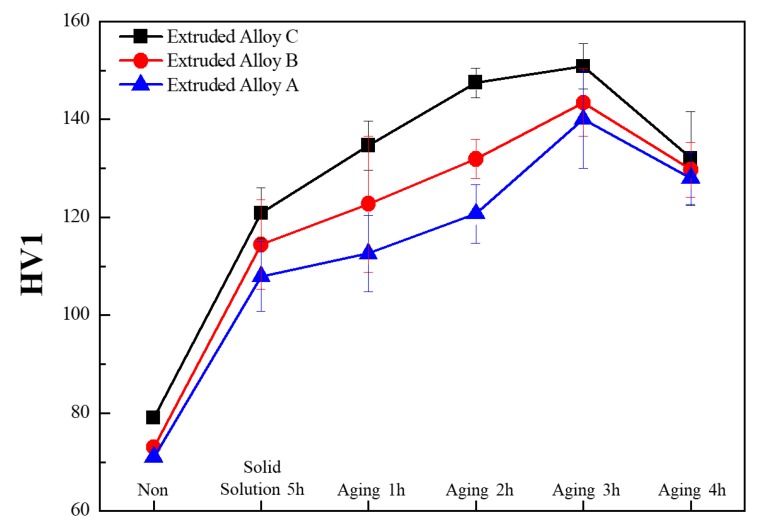
HV1 values of Al-XSi-3Cu-0.6Mg-0.5Fe [X = (A): 10; (B): 12.6; (C): 14] extruded alloys with and without T6 heat treatment.

Figure 3 shows the micro Vickers hardness values (HV1) of Al-Si extruded bars. The HV1 values of Al-Si extruded alloys slightly increased with silicon content increase, and the recorded values were 71, 73, 79 HV1, respectively. The increase in hardness was due to the enhancement of the Al-Si and silicon phases. It is also presumed that, since silicon has a low solubility in aluminium, up to this point it precipitated as pure silicon which is hard and therefore increased the alloy’s hardness [11].

**Figure 4:**
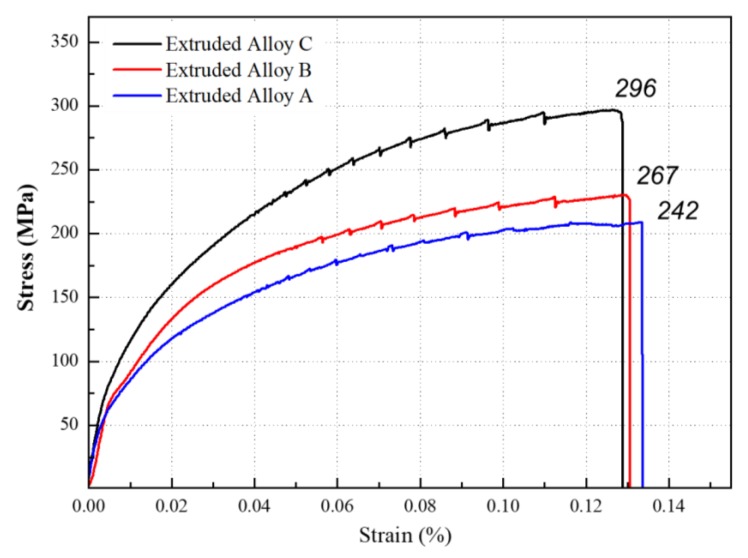
Comparison of tensile strength between the extruded Al-XSi-3Cu-0.6Mg-0.5Fe [X = (A): 10, (B): 12.6, (C): 14] alloys before heat treatment.

One of the important mechanical properties that should be consider for Al-Si alloys is the tensile strength. An increment of the tensile strength value would allow the Al-Si applications. In this work, we measured the tensile strength of the extruded alloys before and after T6 heat treatment. Figure 4 shows the tensile strength of Al-Si alloys measured at room temperature. Before T6 heat treatment, the tensile strength increased, and the elongation rate decreased simultaneously by increasing the silicon content. The values obtained were 242, 267, and 296 MPa for alloy A, alloy Band alloy C, respectively, simultaneously, the elongation rates observed were 13.3, 13, and 12.8%, respectively.

It was presumed that the Al-Si interface was enhanced by increasing the Si percentage. When stress was applied during the tensile test, the soft aluminium phase deforms before the hard silicon phase, triggering dislocations in the Al matrix [6]. As the dislocations moves towards the Al-Si interface, the silicon particles restrict the movement. Hence, it should be possible to apply more stress in the presence of a high silicon percentage, overcoming dislocations which cause plastic deformation. Therefore, the tensile strength was improved by increasing the silicon content. The decrement of the elongation rates with the increase of the silicon weight percentage was due to the plastically brittle nature of silicon in the matrix and the cumulative volume of the hard silicon phase.

### 3.3. SEM Analysis of Tensile Fractured Surfaces Without T6 heat Treatment

**Figure 5:**
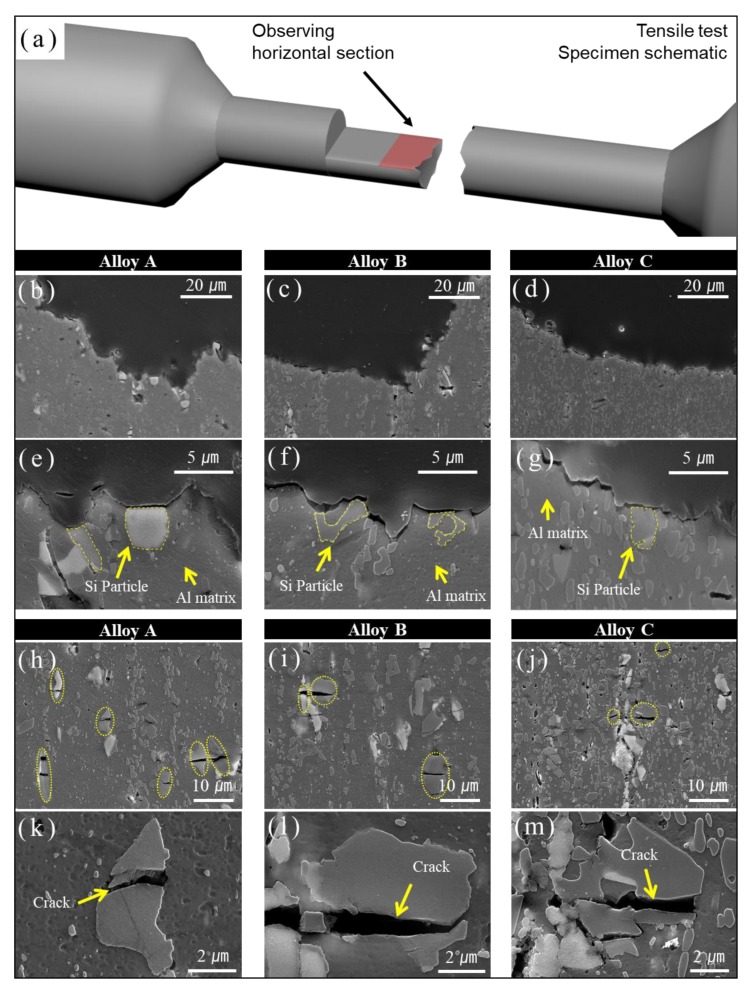
(**a**) Schematic diagram of the fracture mechanism in the tensile test of specimens of Al-XSi-3Cu-0.6Mg-0.5Fe [X = 10, 12.6, 14] extruded alloy; (**b**–**d**) show low-magnification images and (**e**–**g**) show high-magnification images of the fracture surface at the boundary of the specimens; (**h**–**j**) show low-magnification images and (**k**–**m**) show high-magnification images of the fracture surface at the center of the specimens.

Figure 5 displays the SEM morphology and cross-sectional microstructure of the fractured tensile specimens before heat treatment. Figure 5a shows the schematic diagram of fractured tensile specimens’ surfaces and illustrates how the microstructure was examined. The morphology and microstructure of the cracked portion (red-shaded portion in Figure 5a) in the three samples are shown in Figure 5b–g. It is observed from Figure 5e–g that the coarse silicon decreased, and the fine silicon phase tended to increase by increasing silicon content. The marginal increment of silicon phases with silicon content was due to the Lever rule [11]. As a result, the mechanical properties, such as tensile strength and hardness, gradually increased in alloy C compared to alloy A and alloy B.

As seen in Figure 5e–g, the silicon phases which were distributed in the Al matrix showed dissimilar shapes, i.e., roundish, spheroid, and ligament.

Figure 5h–m shows the morphology and microstructure in the middle of the tensile specimens. As seen in Figure 5h–m, the crack was differentially located in Si-phases with different shapes. The crack in all primary silicon phases was an indication of good interfacial bonding arising between the primary silicon particles and the aluminium matrix [11], which was attributed to the significant refining effect of hot extrusion. By applying a force through the tensile test, cracks gradually started in the Si phases. There was a fluctuation in the tensile strength signal in the extruded alloys. This Si phase fracture mechanism is consistent with the report by Hong et al. [5] that the ruin and failure of Al-Si alloys are in general related to development of cracks in the primary Si phase.

### 3.4. Influence of T6 Heat Treatment on Al-Si Extruded Alloys

**Figure 6:**
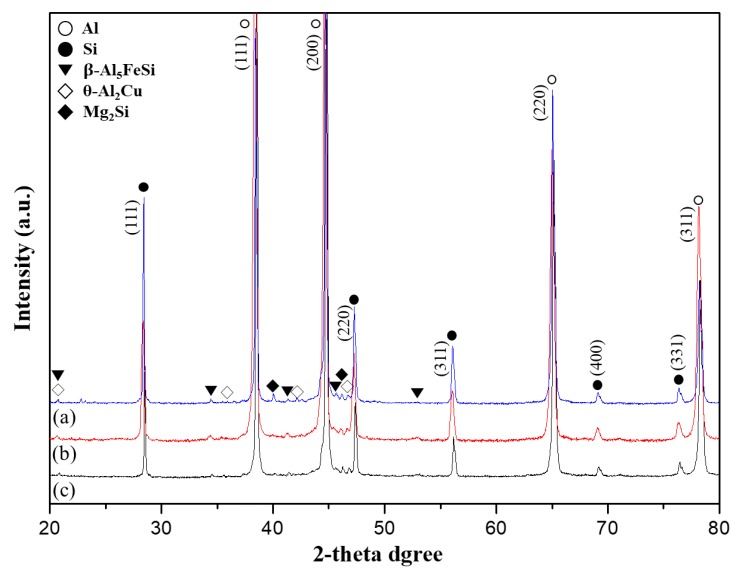
XRD patterns of Al-XSi-3Cu-0.6Mg-0.5Fe [X = (**a**): 10; (**b**): 12.6; (**c**): 14] extruded bars after T6 treatment.

Figure 6 shows the XRD data analysis of T6 heat-treated Al-Si alloys. As the silicon content was increased, the peak position of the silicon phase also increased, and other few peaks, like those of the Mg_2_Si, *θ*-Al_2_Cu, and β-Al_5_FeSi phases, were also noticed. From Figure 3d and the XRD analysis, it was presumed that the modification of the (β-Al_5_FeSi) phase did not occur even after the T6 heat treatment. Thus, the T6 heat treatment showed a negligible effect on the β-Al_5_FeSi phase, which was also observed by Costa et al. [2] and Wu et al. [23]. Oxygen discrete peaks were not observed, since the oxygen content was in a very narrow range, undetectable by X-ray analysis.

The HV1 values of the heat-treated Al-Si alloys are shown in Figure 3. After solution heat treatment and quenching of the solution, the hardness increased by 50% in comparison with the extruded alloys, and the recorded values were 108 HV1, 114 HV1, 120 HV1 for alloy A, alloy B, and alloy C, respectively. This was due to the formation of precipitates which enhanced the hardness. Here, we selected a solution heat treatment of 500 °C for 5 h, because, at a high temperature and with a long time, inceptive melting of *θ*-Al_2_Cu, due to high thermal stress, occurred after quenching, which prompted a deterioration of the mechanical properties [7,12,22]. We affirm that our solution treatment showed effective strengthening and, as shown in the literature [22], the addition of Mg to an Al-Si-Cu alloy showed a higher strengthening effect at 500 °C than at 480 °C. In addition, Costa et al. [2] compared treatments for 5 and 8 h and demonstrated that 5 h was effective in modifying the alloy microstructure, which resulted in improved hardness. After solution heat treatment and quenching, age-hardening was validated as an important process. Choosing the proper aging time and temperature is essential to avoid deleterious effects to the mechanical properties of aluminium alloys. In this prospect, we optimized the proper aging time with respect to hardness and performed the Vickers hardness test for different aging periods (1, 2, 3, and 4 h). This kind of experimental procedure was also performed by Gupta et al. [11] to obtain the proper peak aging time, and their results showed a high hardness value after 9 h of aging.

As shown in Figure 3, the maximum hardness was obtained at 3 h with 140, 143, 150 HV1 for alloy A, alloy B, and alloy C, respectively. With a further heat treatment for 4 h, the hardness tended to decrease, reaching values of 128, 129, 132 HV1. From these results, it was concluded that a 4 h treatment caused over-aging characterized by particle agglomeration, leading to the deterioration of strength and hardness. A treatment of 3 h was considered a proper aging time to enhance the properties of aluminium alloys through the formation of coherent precipitates capable to cutoff aluminium dislocations [7].

**Figure 7:**
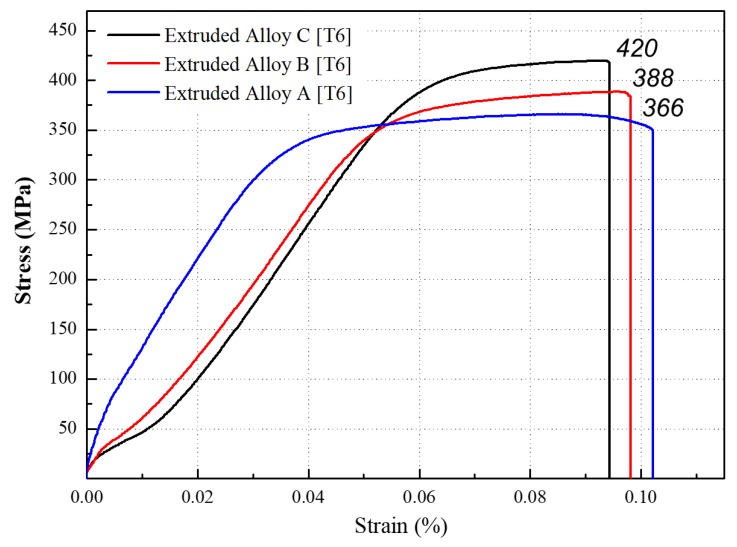
Comparison of tensile strength between the Al-XSi-3Cu-0.6Mg-0.5Fe [X = (a): 10, (b): 12.6, (c): 14] extruded alloys after T6 heat treatment.

Figure 7 shows the tensile strength values of the T6 heat-treated samples. The tensile strength was greatly enhanced after T6 heat treatment by meticulously choosing the optimum aging time as 3 h. In comparison to the extruded alloys that did not undergo heat treatment, the tensile strength was improved by ~45 % in all T6 extruded samples. The recorded tensile strength values were 366, 388, and 420 MPa for alloy A, B, C, respectively, and the elongation rates were 10, 9.7, and 9.4 %. The obtained tensile strength of 420 MPa was greater than those previously reported by Wu et al. [23] and Ke et al. [8].

**Figure 8:**
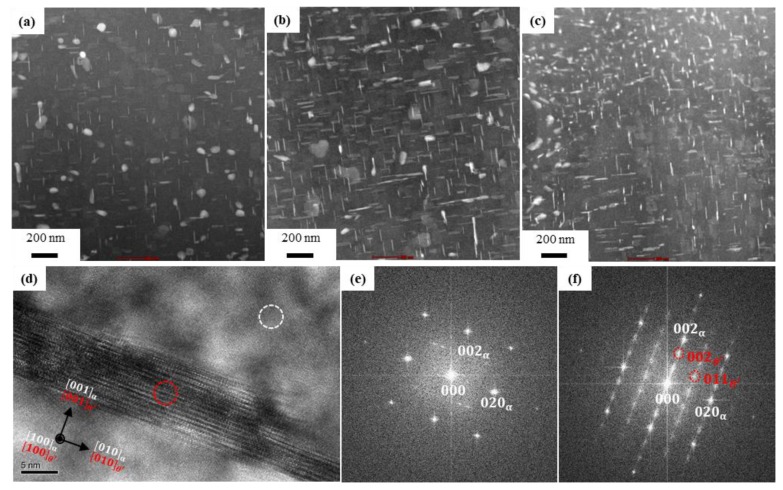
Transmission electron microscope (TEM) image of Al-XSi-3Cu-0.6Mg-0.5Fe [X = (**a**): 10, (**b**): 12.6; (**c**): 14] T6 heat-treated extruded bars; (**d**) high-resolution TEM (HRTEM) image of *θ*′-Al_2_Cu phase. Fast Fourier transform pattern of the (**e**) white and (**f**) red circles displayed in the HRTEM image shown in (**d**).

Figure 8 shows the TEM analysis of T6 heat-treated extruded alloys. From Figure 8a–c TEM images, it is evident that there are numerous white spots and lines in the Al matrix. In general, the age-hardening response is very efficient in the Al-Si-Cu-Mg alloy [7], and it was shown that copper has a great influence at high temperatures, increasing the strength of Al-Si alloys through the dispersion of the Al_2_Cu phase in the Al matrix. As shown in Figure 8a–c, the Al_2_Cu phase was finely dispersed at different locations in a definite pattern by meticulously selecting proper aging time and temperature.

Figure 8d shows the high-resolution transmission electron microscope (HRTEM) image of *θ*’-Al_2_Cu precipitated with streaks, as observed in Figure 8c. The direction of the electron beam was parallel to ${\left[100\right]}_{Al,\;\theta^{\prime }}$. The fast Fourier transform (FFT) patterns in Figure 8e,f were obtained from the regions delimited by the white and red circles in Figure 8d, corresponding to the matrix, *α*, and *θ*′ phase, respectively. The FFT pattern confirmed the existence of the *θ*′-Al_2_Cu phase along the ${\left[001\right]}_{\theta^{\prime }}$ direction. The orientation relationship between the matrix *α* and *θ*′ is known to be ${\left(001\right)}_{\alpha \;}⫽{\left(001\right)}_{\theta^{\prime }\;},{\left[001\right]}_{\alpha \;}⫽{\left[001\right]}_{\theta^{\prime }}$.

It was observed that the Al_2_Cu phases increased with Si content in the Al matrix, which is shown in Figure 8a–c. The increment in the Al_2_Cu phases with the increase of Si wt.% was interpreted as due to the increase by 20-fold of the diffusion coefficient of copper in aluminium, following the increase of silicon content in the alloy [7]. In Al-Si alloys, Mg can predominantly control and reduce the formation of Al_2_Cu phases [23], and this effect was compensated by increasing the silicon content, which increases the formation of the Al_2_Cu phases.

In this Al-Si-Cu-Fe-Mg composition, copper (3 wt.%) and magnesium (0.6 wt.%) were added for the precipitation hardening effect. According to Gowri et al. [20], a minimum 3 wt.% of copper must be included in Al-Si-Cu-Fe-Mg alloys for the precipitation of Mg_2_Si and Al_2_Cu phases. Caceres et al. [24] proposed that, typically, 0.3 to 0.7 wt.% of Mg in Al-Si foundry alloys precipitates as Mg_2_Si, and, upon T6 heat treatment, this Mg phase can dissolve in the solid solution and acts as an effective strengthening precipitate [7]. As shown in Figure 6, the XRD analysis confirmed the presence of the Mg_2_Si phase in alloy A, alloy B, and alloy C, and it was assumed that the Mg_2_Si phase also would act as a strengthening precipitate.

Hence, after T6 heat treatment, the mechanical properties were improved as a result of the effective combination of the Mg_2_Si, Al_2_Cu phase with the streak-like shape phase (*θ*′ phase), which were dispersed in the Al matrix, and plausibly the transformation of the Si morphology leading to a strengthening phase that could precipitate during the heat treatment [15]. The effective combination of the Al_2_Cu phase precipitated during heat treatment and the hard silicon slowed the aluminium dislocations [25], and, consequently, the elongation rate decreased progressively compared to the non-heat-treated alloys.

## 4. Conclusions

In this study, Al-XSi-3Cu-0.5Fe-0.6Mg (X = 10, 12.6, 14) alloys were prepared using the die casting and extrusion technique. The effect of Si content on the microstructure and mechanical properties of the extruded bar and of the T6 heat-treated specimens were systematically investigated. The following conclusions were obtained from the experimental results:

1. The Si phase was finely distributed in the Al matrix along the extrusion direction, and the amount of Si phase increased with the increase of Si content. Cracks in the Si phases and fragmented intermetallic (β-Al_5_FeSi) phase were observed in the Al matrix.
2. The tensile strength of the extruded bar improved with the increase of Si content, while the ductility decreased. The maximum tensile value obtained was 296 MPa for the 14 wt.% Si-doped sample.
3. To further improve the tensile strength, T6 heat treatment was carried out. The optimum aging time was confirmed by the Vickers hardness values. The HV1 value increased after up to 3 h of aging and reached a maximum value of 150 HV1.
4. The TEM images of T6 heated specimens displayed a coherent Al_2_Cu phase in the Al matrix. A precipitated *θ*′-Al_2_Cu phase was confirmed by the FFT patterns.
5. The tensile strength improved by ~45 % compared to non-heat-treated alloys. A maximum tensile strength of 420 MPa was obtained for the Al-14Si-3Cu-0.5Fe-0.6Mg alloy, due to the formation of the *θ*′-Al_2_Cu phase in the Al matrix.

## Figures and Tables

**Table 1:** Nominal chemical composition of Al-XSi-3Cu-0.6Mg-0.5Fe [X = (a): 10 (Alloy A), (b): 12.6 (Alloy B), (c): 14 (Alloy C)] cast alloys.

Alloy	Composition (wt.%)					Density of Casting Alloy (g/cm^3^)
	Si	Cu	Mg	Fe	Al	
4007A	9.0~10.5	2.5~3.5	0.5~0.7	<0.5	Bal.	-
Alloy A	10	3	0.6	0.5	Bal.	2.715
Alloy B	12.6	3	0.6	0.5	Bal.	2.701
Alloy C	14	3	0.6	0.5	Bal.	2.688
